# Association of Nutrition Education and Its Interaction with Lifestyle Factors on Kidney Function Parameters and Cardiovascular Risk Factors among Chronic Kidney Disease Patients in Taiwan

**DOI:** 10.3390/nu13020298

**Published:** 2021-01-21

**Authors:** Adi-Lukas Kurniawan, Ya-Lan Yang, Mei-Yun Chin, Chien-Yeh Hsu, Rathi Paramastri, Hsiu-An Lee, Po-Yuan Ni, Jane Chao

**Affiliations:** 1 School of Nutrition and Health Sciences, College of Nutrition, Taipei Medical University, 250 Wu-Hsing Street, Xinyi District, Taipei 110, Taiwan; 8lukas@ntunhs.edu.tw (A.-L.K.); rara.paramastri@gmail.com (R.P.); a10011352@yahoo.com.tw (P.-Y.N.); 2 Research Center for Healthcare Industry Innovation, National Taipei University of Nursing and Health Sciences, 365 Ming-Te Road, Peitou District, Taipei 112, Taiwan; 3 Diet and Nutrition Department, Shuang Ho Hospital, Taipei Medical University, 291 Jhongjheng Road, Jhonghe District, New Taipei 235, Taiwan; 70735@s.tmu.edu.tw; 4 Department of Information Management, National Taipei University of Nursing and Health Sciences, 365 Ming-Te Road, Peitou District, Taipei 112, Taiwan; cyhsu@ntunhs.edu.tw; 5 Master Program in Global Health and Development, College of Public Health, Taipei Medical University, 250 Wu-Hsing Street, Xinyi District, Taipei 110, Taiwan; 6 Department of Computer Science and Information Engineering, Tamkang University, 151 Yingzhuan Road, Tamsui District, New Taipei 251, Taiwan; billy72325@gmail.com; 7 Nutrition Research Center, Taipei Medical University Hospital, 252 Wu-Hsing Street, Xinyi District, Taipei 110, Taiwan

**Keywords:** nutrition education, lifestyle factors, kidney function, cardiovascular disease, chronic kidney disease

## Abstract

We evaluated the interactive effects of nutrition education (NE) and lifestyle factors on kidney function parameters and cardiovascular risk factors among chronic kidney disease (CKD) patients. This cross-sectional cohort study recruited 2176 CKD stages 3–5 patients aged > 20 years from Integrated Chronic Kidney Disease Care Network, Shuang Ho Hospital, Taiwan between December 2008 and April 2019. The multivariable regression analysis was performed to investigate the interactive effects of NE with lifestyle factors on kidney function parameters and cardiovascular risk factors. Relative excess risk due to interaction (RERI) and attributable proportion (AP) were applied to assess additive interaction. Patients who were smoking or physically inactive but received NE had better estimated glomerular filtration rate (eGFR) (β: 3.83, 95% CI: 1.17–6.49 or β: 3.67, 95% CI: 2.04–5.29) compared to those without NE. Patients with smoking and NE significantly reduced risks for having high glycated hemoglobin A_1c_ (HbA_1c_) by 47%, high low-density lipoprotein cholesterol (LDL-C) by 38%, and high corrected calcium (C-Ca) by 50% compared to those without NE. Moreover, NE and smoking or inactive physical activity exhibited an excess risk of high C-Ca (RERI: 0.47, 95% CI: 0.09–0.85 for smoking or RERI: 0.46, 95% CI: 0.01–0.90 and AP: 0.51, 95% CI: 0.03–0.99 for physical activity). Our study suggests that CKD patients who were enrolled in the NE program had better kidney function. Thus, NE could be associated with slowing kidney function decline and improving cardiovascular risk factors.

## 1. Introduction

Over the past 10 years, chronic kidney disease (CKD) has become the top 10 leading causes of death in Taiwan [1]. According to the United States Renal Data System report in 2018, Taiwan had been recorded to have the greatest prevalence and incidence of end-stage renal disease (ESRD) [2]. A recent study in Taiwan showed that the overall prevalence of CKD stages 1 to 5 was 15.5% and 9.1% for CKD stages 3 to 5 with an incidence of nearly 27.2 per 1000 people per year [3]. Moreover, according to Taiwan’s national health insurance statistics in 2018, CKD was the first place for the medical expenses of the national health insurance, indicating that CKD not only threatens population health but also becomes a serious financial burden of national medical resources [4].

It is well known that CKD patients are prone to develop cardiovascular disease (CVD) including stroke, heart failure, and myocardial infarction, as well as increase the risk for ESRD progression [5,6]. Thus, the increased risk of death in CKD patients was largely attributable to CVD [7]. A 13-year cohort study in Taiwan reported that CKD patients had 83% higher mortality for all-cause (HR: 1.83, 95% CI: 1.73–1.93) and 100% higher for CVD (HR: 2.00, 95% CI: 1.78–2.25) [8]. Diabetes, abnormal lipid, calcium, and phosphorus metabolism have been known as major risks for developing CVD in CKD patients [9,10]. Abnormal calcium and phosphorus levels may also generate vascular calcification and other cardiovascular events [11,12].

Treatment for ameliorating comorbid conditions, slowing the progression of kidney failure, malnutrition, and mineral-related bone disease, and minimizing the risk for CVD should begin at the early stage of CKD [13]. Thus, a balance between clinical and nutritional therapy is an advisable intervention to provide a better quality of life in CKD patients [14]. In this regard, not only dietary intervention but also nutrition education (NE) or dietary counseling may play an important role to ensure an optimal nutrition status and preserve renal function in CKD patients. However, the majority of previous studies in an educational intervention aimed to improve nutritional status, adherence to a diet, and quality of life [14,15,16,17]. The studies investigating the role of NE in kidney function parameters and cardiovascular risk factors as well as the interactive effects of NE and lifestyle behaviors are also scarce in the literature. Moreover, unhealthy lifestyle behaviors including smoking, alcohol drinking, and low physical activity are closely correlated to CKD and CVD [18,19,20]. We hypothesized that the NE program as a supporting therapy can help to slow kidney function decline and improve cardiovascular risk factors in CKD patients. Therefore, we aimed to explore the interactive effects of NE and lifestyle factors on kidney function parameters and cardiovascular risk factors among CKD patients.

## 2. Materials and Methods

### 2.1. Study Subjects and Nutrition Education

This study was a cross-sectional approach and collected CKD patients at stages 3–5 with proteinuria from the pre-ESRD program referred by the Department of Nephrology at Shuang Ho Hospital, Taipei Medical University, Taiwan between December 2008 and April 2019. We collected the data of CKD patients from the “Integrated Chronic Kidney Disease Care Network,” which has been developed for more than 10 years in the hospital in Taiwan. The collected data included sociodemographic information, lifestyle, medical records, anthropometric data, and biochemical parameters. Moreover, CKD patients were referred by the case manager to register for having NE individually by the dietitian in the hospital. Patients were given at least 1–2 NE sessions within a year, and more NE sessions if kidney function parameters were worse after follow-up every 3 months. The duration for each NE session was 30–60 min. The content of NE at the first counseling session included the evaluation of dietary history, dietary intake for energy, macronutrients, and micronutrients, and nutritional status in CKD patients and the recommendation of general dietary guidelines for CKD based on weight status and CKD stage of the patients for at least 30 min. After the first session of the NE program, an individual diet plan and nutrient intakes specific for protein, phosphorus, potassium, sodium, and water were recommended. Dietary intake was modified and monitored individually according to the physiological and nutritional status of CKD patients.

**Figure 1:**
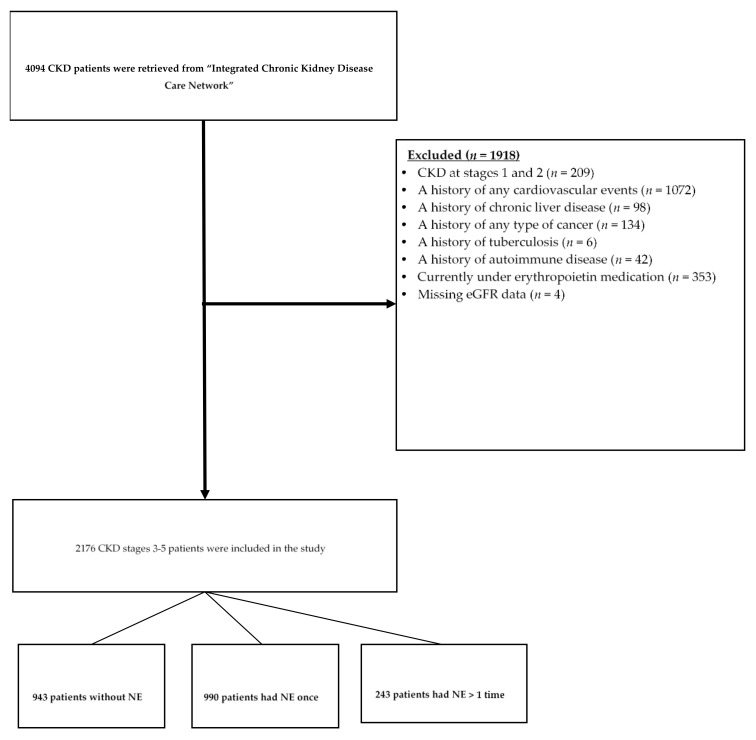
Flowchart diagram of CKD patients’ selection. CKD: chronic kidney disease, eGFR: estimated glomerular filtration rate, NE: nutrition education.

Initially, 4094 CKD patients registered in the pre-ESRD program for NE between December 2008 and April 2019. After excluding CKD patients at stages 1 and 2 (*n* = 209) and those who had a history of cardiovascular disease including congestive heart disease, ischemic heart disease, and cerebral vascular disease (*n* = 1072), chronic liver disease (*n* = 98), cancer (*n* = 134), tuberculosis (*n* = 6), and autoimmune disease (*n* = 42), erythropoietin therapy (*n =* 353), or missing estimated glomerular filtration rate (eGFR) data (*n* = 4), a total of 2176 CKD stages of 3–5 patients aged > 20 years old were included in the analysis. Among 2176 CKD patients, 943 patients had no NE, 990 patients only had NE once, and 243 patients had NE more than once (Figure 1). We excluded CKD patients with a history of any cardiovascular event or other chronic diseases because these disease events may become strong confounders to interfere with the outcomes of this study. Erythropoietin treatment may have potential effects on cardiovascular disease [21] and, further, affect the outcomes of cardiovascular risk factors. Prior to the NE program, all CKD patients signed written informed consent, and personal information was kept confidential. The Taipei Medical University Joint Institutional Review Board (N202001055) approved this study.

### 2.2. Sociodemographic Data, Lifestyle, and Use of Drugs

Sociodemographic data including age, gender, education (below high school level and high school level or above), marital status (married and unmarried including those who divorced, widowed, and separated), and occupation (e.g., civil servant, labor, businessman, housekeeper, farmer, teacher, retirement, etc.) were collected from the database. Lifestyle including smoking, drinking alcohol, or chewing betel nuts was collected on a daily basis in the past. Smoking and drinking status were classified as ‘yes’ if CKD patients smoked a cigarette or drank alcohol daily and as ‘no’ if otherwise. Physical activity data including the type (e.g., regular walking, fast walking, jogging, dance, gymnastics, biking, hiking, or other), frequency (no, 1–2 times/week, 3–4 times/week, 5–6 times/week, or ≥7 times/week), and duration (no, <30 min, 30–60 min, 60–90 min, and >90 min) were recorded. Patients having physical activity ≥30 min/week were classified as physically ‘active’ or ‘inactive’ if otherwise. The use of drugs including diuretics, angiotensin II receptor blocker, angiotensin-converting enzyme inhibitor, calcium channel blocker, calcium-phosphorus binder, iron supplementation, antihypertensive agent, hypolipidemic agent, hypoglycemic agent, and insulin injection were collected.

### 2.3. Clinical and Biochemical Data

Blood pressure and biochemical data were collected before the entry to the pre-ESRD program and followed up every three months. Body weight was measured when patients visited the dietitian for NE sessions. The clinical and biochemical data were analyzed using these closest to the last NE session. Body weight and height were measured by using an auto-anthropometers (AHS 700, Kaohsiung, Taiwan). Body mass index (BMI) was calculated by weight (kg) divided by the square of height (m^2^). Systolic and diastolic blood pressure (BP) was measured by using an oscillometric machine (OMRON HBP-9020, Taipei, Taiwan). Fasting blood glucose (FBG), albumin, triglycerides (TG), total cholesterol (TC), high-density lipoprotein cholesterol (HDL-C), low-density lipoprotein cholesterol (LDL-C), calcium (Ca), phosphorus (P), blood urea nitrogen (BUN), serum creatinine, urine protein, and urine creatinine were measured by an auto-chemical analyzer (Beckman DxC 800, California, USA). Glycated hemoglobin A_1c_ (HbA_1c_) was measured by capillary electrophoresis (Sebia II, Lisses, France). Serum calcium levels were corrected for serum albumin by using Payne’s formula: corrected calcium (C-Ca) (mmol/L) = calcium (mmol/L) + 0.02 × [40 − serum albumin (g/L)] [22]. Cardiovascular risk factors were defined as follows: high FBG if ≥5.56 mmol/L (100 mg/dL), high HbA_1c_ if ≥5.7%, high TG if ≥1.70 mmol/L (150 mg/dL), high TC if ≥5.18 mmol/L (200 mg/dL), low HDL-C if ≤1.04 mmol/L (40 mg/dL), and high LDL-C if ≥2.59 mmol/L (100 mg/dL) [19,23]. High C-Ca was defined as serum levels of corrected calcium ≥2.37 mmol/L (9.5 mg/dL), and high P was defined as serum levels of phosphorus ≥1.49 mmol/L (4.6 mg/dL) based on National Kidney Foundation guidelines [24]. The value of eGFR was calculated by using the Modification of Diet in Renal Disease study equation [25]. Moreover, based on eGFR levels, the stages of CKD were classified into: CKD stages 3a (45–59 mL/min/1.73 m^2^), 3b (30–44 mL/min/1.73 m^2^), 4 (15–29 mL/min/1.73 m^2^), and 5 (<15 mL/min/1.73 m^2^).

### 2.4. Statistical Analysis

This cross-sectional cohort study sampled CKD patients cross-sectionally, and then retrospectively evaluated the history of nutrition education and outcomes over a specified time period. The characteristics of study patients were compared between those who, with and without NE, used the chi-square test for categorical data (expressed as number and percentage) and Wilcoxon rank-sum test for continuous data (expressed as a median and interquartile range due to a non-normal distribution). In the cross-sectional analysis, the explanatory regression model or multivariable regression model was used for identifying variables that had a scientifically meaningful and statistically significant relationship with an outcome. A linear regression analysis was used to assess the association between NE and its interaction with lifestyle and kidney function parameters. The data are expressed as beta (β) coefficient and 95% confidence intervals (CI). Meanwhile, the cross-sectional study with binary outcomes was analyzed by logistic regression to investigate the association between NE and its interaction with lifestyle and cardiovascular risk factors. The data are expressed as odds ratio (OR) and 95% CI. To evaluate additive interaction in the joint effect of 2 predictor factors from multiplicative models and estimate the excess risk ratio from the ORs, we used relative excess risk due to the interaction (RERI), which was also referred to as the interaction contrast ratio (ICR) without exposure and attributable proportion due to an interaction (AP) with both exposures. Detailed information on an additive interaction has been published elsewhere [26,27]. In the analysis of RERI and AP, predictor factors in the interaction and outcomes were dichotomized into with or without exposure and with or without an interaction effect, respectively. RERI is calculated by the formula: RR_A+B+_ − RR_A+B-_ − RR_A-B+_ + 1, where RR indicates relative risk referring to OR in a logistic regression model, A or B represents the predictor factor in the interaction, and + or − means with or without exposure [26]. While AP is calculated by the formula: RERI/RR_A+B+_ [26,27]. RERI or AP = 0 indicates no interaction, RERI or AP > 0 means positive interaction or more than additivity, and RERI or AP < 0 represents a negative interaction or less than additivity [27]. All analyses were adjusted for age, gender, education, marital status, occupation, smoking, drinking alcohol, chewing betel nut, physical activity, use of diuretics and other drugs, BMI, systolic BP, and diastolic BP. The ‘ic [outcome] [predictor A] [predictor B], rrby(or)’ command was used to estimate the additive interaction effect and the 95% CIs. All the statistical analyses were performed by using STATA version 13 (STATA Corp LLC, College Station, USA), and *p* < 0.05 was considered statistically significant.

## 3. Results

### 3.1. Characteristics, Clinical Data, and Biochemical Measures of the Study Patients

The data of 2176 CKD stages 3–5 patients were retrieved after removing those who met the exclusion criteria, as mentioned in Figure 1. Among 2176 CKD patients, 943 (43.3%) patients did not enroll in the NE program, while 990 patients (45.5%) had NE once and 243 (11.2%) patients had NE more than once. We pooled CKD patients with NE regardless of the frequency of NE for comparisons. Table 1 presents the characteristics of the study patients (*n* = 2176). Patients with NE were younger (*p* < 0.001), physically active (*p* = 0.002), and had lower proportions of low education (*p* < 0.001) and diuretic drug users (*p* = 0.004) compared to those without NE. The clinical and biochemical data of CKD patients with or without NE are shown in Table 2. Patients with NE had higher values of BMI (*p* = 0.012) and albumin (*p* = 0.002), but lower values of HbA_1c_ (*p* < 0.001) and LDL-C (*p* = 0.005). In terms of kidney function parameters, CKD patients with NE had lower BUN levels (*p* = 0.001), serum creatinine levels (*p* = 0.04), and urine PCR (*p* = 0.019), but higher eGFR (*p* = 0.003) and urine protein (*p* < 0.001) compared to those without NE. The percentage of stage 5 CKD with and without NE was 15.7% and 28.3%, respectively.

### 3.2. Nutrition Education and Kidney Function

The adjusted beta (β) coefficients of kidney function parameters by NE and its interaction with lifestyle factors are indicated in Table 3. The adjusted model showed that CKD patients with NE significantly increased eGFR values by 2.9 mL/min/1.73 m^2^ (*p* < 0.01), and decreased BUN by 4.2 mmol/L (*p* < 0.01), serum creatinine by 122.8 μmol/L (*p* < 0.01), urine creatinine by 1.5 mmol/L (*p* < 0.01), and urine PCR by 40.2 mg/mmol (*p* < 0.05). However, there was no significant association between NE and urine protein. Moreover, the fully adjusted model revealed that CKD patients without drinking significantly decreased urine creatinine by 0.8 mmol/L (95% CI: −1.61 to −0.05, *p* < 0.05) compared to those with drinking (Supplementary Table S1). Compared to those who were physically inactive, CKD patients who were physically active had significantly higher eGFR values (β: 2.11, 95% CI: 0.62–3.60, *p* = 0.006) and lower BUN (β: −1.34, 95% CI: −2.41 to −0.27, *p* = 0.012), serum creatinine levels (β: −30.48, 95% CI: −56.00 to −4.96, *p* = 0.017), and urine PCR (β: −37.73, 95% CI: −74.65 to −0.82, *p* = 0.045 (Supplementary Table S1). However, there was no correlation between smoking status and all kidney function parameters.

Additionally, the interaction analysis showed that CKD patients with NE no matter smoking status were more likely to have better kidney function parameters (Table 3). Patients with NE and smoking had significantly higher eGFR values (β: 3.83, 95% CI: 1.17–6.49, *p* = 0.005) and lower BUN (β: −4.52, 95% CI: −6.40 to −2.63, *p* < 0.01), serum creatinine (β: −168.79, 95% CI: −213.13 to −124.44, *p* < 0.01), urine creatinine (β: −2.33, 95% CI: −3.27 to −1.38, *p* < 0.01), and urine PCR (β: −78.21, 95% CI: −143.46 to −12.95, *p* < 0.05) than those without NE but smoking. Patients with NE and non−drinking significantly increased eGFR values by 4.3 mL/min/1.73 m^2^ (*p* < 0.05) and decreased serum creatinine by 83.9 μmol/L (*p* < 0.01) and urine creatinine by 3.2 mmol/L (*p* < 0.01) compared to those who drank and without NE. In contrast, CKD patients who did not drink and without NE had lower eGFR levels (β: −4.40, 95% CI: −7.60 to −1.20, *p* = 0.007) compared to those who drank and without NE. Patients with NE and active physical activity were also more likely to have better kidney function parameters. Patients with NE and active physical activity significantly increased eGFR values by 4.7 mL/min/1.73 m^2^ (*p* < 0.01), and decreased BUN by 5.3 mmol/L (*p* < 0.01), serum creatinine by 144.5 μmol/L (*p* < 0.01), urine creatinine by 1.5 mmol/L (*p* < 0.01), and urine PCR by 74.1 mg/mmol (*p* < 0.01). CKD patients who were physically active but without NE also significantly increased eGFR values by 3.6 mL/min/1.73 m^2^ (*p* = 0.003) compared to those who were physically inactive and without NE.

### 3.3. Nutrition Education and Cardiovascular Risk Factors

Table 4 indicates the adjusted ORs of cardiovascular risk factors by NE and its interaction with lifestyle factors. Patients with NE were less likely to have high HbA_1c_ (OR: 0.61, 95% CI: 0.45–0.82, *p* = 0.001), high TG (OR: 0.72, 95% CI: 0.57–0.89, *p* = 0.003), high LDL-C (OR: 0.76, 95% CI: 0.60–0.96, *p* = 0.019), high P (OR: 0.76, 95% CI: 0.59–0.98, *p* = 0.033), and low HDL-C (OR: 0.68, 95% CI: 0.48–0.98, *p* = 0.038). Furthermore, the fully adjusted model revealed that there were no significant associations between all lifestyle factors and cardiovascular risk factors, except for a reduced risk of having high P in CKD patients who were physically active (OR: 0.73, 95% CI: 0.55–0.96, *p* = 0.026) (Supplementary Table S2).

The adjusted model of interaction analysis showed that CKD patients with NE but non-smoking were less likely to have low HDL-C (OR: 0.49, 95% CI: 0.26–0.92, *p* = 0.027), high C-Ca (OR: 0.55, 95% CI: 0.32–0.98, *p* = 0.037), and high P (OR: 0.54; 95% CI: 0.35–0.84, *p* = 0.007) compared to those with smoking but without NE (Table 4). Patients with smoking and NE had reduced risks of having high HbA_1c_ (OR: 0.53, 95% CI: 0.30–0.96, *p* = 0.035), high LDL-C (OR: 0.62, 95% CI: 0.40–0.97, *p* = 0.037), and high C-Ca (OR: 0.50, 95% CI: 0.27–0.94, *p* = 0.03) compared to those with smoking but without NE. Moreover, NE and smoking had only an excess risk of high C-Ca (RERI: 0.47, 95% CI: 0.09–0.85, *p* = 0.015), while there were no excess risks in other cardiovascular risk factors. Patients with drinking and NE also had reduced risks of having high HbA_1c_ (OR: 0.40, 95% CI: 0.17–0.94, *p* = 0.035) and high LDL-C (OR: 0.53, 95% CI: 0.32-0.90, *p* = 0.018) compared to those with drinking but without NE. There were no significant additive risks between NE and drinking alcohol in all cardiovascular risk factors (RERI *p* > 0.05). Compared to those who were physically inactive and without NE, CKD patients who were physically inactive but with NE decreased risks of having high HbA_1c_ by 42% (OR: 0.58, *p* = 0.005), high TG by 31% (OR: 0.69, *p* = 0.006), high LDL-C by 28% (OR: 0.72, *p* = 0.025), high C-Ca by 31% (OR: 0.69, *p* = 0.048), and high P by 30% (OR: 0.70, *p* = 0.017). Additionally, CKD patients who were physically active with NE had lower risks of having high HbA_1c_ (OR: 0.64, *p* = 0.044), high TG (OR: 0.60, *p* = 0.002), high LDL-C (OR: 0.64, *p* = 0.009), high P (OR: 0.58, *p* = 0.004), and low HDL-C (OR: 0.54, *p* = 0.018). The interaction between physical activity and NE had an excess risk of high C-Ca (RERI: 0.46, *p* = 0.047, AP: 0.51, *p* = 0.036).

## 4. Discussion

Patients with CKD have higher rates to develop CVD mediated by the catabolic state, which progressively occurred at the end-stage CKD [28]. In recent years, NE has been widely investigated as adjunctive nutrition therapy in the prevention and management of chronic diseases, such as CKD [29]. For end-stage CKD patients, a protein-restricted diet is crucial to ameliorate uremic symptoms or complications [30,31] and delay the need for dialysis [31]. Additionally, adequate energy intake and micronutrient consumption are important to maintain appropriate physical activity and decrease CKD-related comorbidity and mortality in CKD patients [32]. Therefore, NE is potentially beneficial to CKD patients for better awareness of nutrition knowledge and self-management of their diet and lifestyle behavior, which may enhance learning and further improve the outcomes of the disease [33]. The current guidelines for the management of CKD suggest that education focuses more on managing risk factors to delay progression and to allow patients to make informed decisions regarding their treatment [33]. Our study found that CKD patients at stages 3–5 with NE had declined BUN and serum creatinine levels and increased eGFR compared to those without NE. In line with the current guidelines, our findings agreed that the NE program provides supporting management to delay CKD progression. Similarly, the previous studies demonstrated that face-to-face NE was associated with decreases in BUN [34,35] and serum creatinine levels [35,36] in hemodialysis patients.

The present study reported that NE was associated with reduced risks for high HbA_1c_, high TG, low HDL-C, high LDL-C, and high phosphorus. The previous evidence showed that two months after e-learning NE intervention, serum sodium, potassium, and phosphorus levels were significantly decreased in hemodialysis patients who received an NE training message sent twice a week to the Telegram messenger for four weeks when compared to the control group who received standard educational practice instead of an NE intervention [37]. Additionally, hemodialysis patients receiving face-to-face NE for 3 months significantly decreased serum creatinine, potassium, and phosphorus levels compared to the control group receiving routine education rather than NE [36]. Similarly, hemodialysis patients significantly reduced serum sodium, potassium, and calcium levels after receiving four 30-min face-to-face NE sessions weekly for 30 days as compared to the baseline before NE intervention [34]. The intervention of NE has been proposed to improve the health outcomes and self-care skills of the patients. The previous study found that the metabolic outcomes were improved in Type 2 diabetic elderly with 10-week 10 NE sessions likely because of strengthening their nutrition knowledge and skills to apply to daily meal planning and health management [38]. Franz et al. [39] suggested that Type 2 diabetic patients who received NE more frequently (1 initial visit + 2 follow-up NE sessions) showed better glycemic control compared to those who only visited a dietitian once for approximately 1 h. Therefore, NE intervention requires sufficient duration and comprehensive NE materials to meet patients’ needs effectively [38]. In addition, a theory-driven nutrition approach can have better learning outcomes by dividing concepts into less information for each NE session to avoid information overload [40].

The development of CKD was associated with unhealthy lifestyle behaviors such as physical inactivity, late-night dinner, and bedtime snacking in middle-aged and older adults [18]. Meanwhile, healthy lifestyle factors, such as non-smoking, moderate or less alcohol drinking, regular physical activity, and a better eating pattern was related to a lower risk of CKD [41]. Therefore, NE is essential to deliver proper knowledge and skills for healthy lifestyle patterns and disease management in CKD patients. Accordingly, combined exposure of NE in CKD patients with unhealthy lifestyle behaviors showed that CKD patients who were smoked or physically inactive but receiving NE exhibited better kidney function parameters and cardiovascular risk factor outcomes, which supports that NE plays a pivotal role in practicing patients’ self-health management.

Our study showed that smoking was associated with neither kidney function parameters nor cardiovascular risk factors in CKD patients after the covariates were fully adjusted. However, the previous study found that current smokers significantly increased the odds ratio of developing CKD compared to non-smokers (OR: 2.18, 95% CI: 1.57–3.03), while current drinkers did not significantly increase the odds ratio of developing CKD compared to non-drinkers (OR: 1.08, 95% CI: 0.54–2.14) [42]. Additionally, the patients with CKD and smoking were more likely to have CVD after being adjusted for age and sex (HR: 2.28, 95% CI: 1.25–4.17) compared to those with neither condition [43]. Alcohol consumption was positively correlated to HDL-C levels in non-drinkers compared to those with the highest alcohol consumption (>7 drinks/week for women and >14 drinks/week for men) [44]. Our study also revealed that drinking alcohol was only negatively correlated with urine creatinine levels but not associated with cardiovascular risk factors in CKD patients after the covariates were fully adjusted. The different results between the present and previous studies might be due to the different study populations and various adjustments of covariates. Both smoking and alcohol consumption could have effects on cardiovascular risk factors and generally did not influence the same risk factors in a similar way [45]. Excessive exposure to combined smoking and alcohol consumption could potentially worsen cardiovascular risk factors [45]. Our results indicated that physical activity was associated with improved kidney function parameters and reduced high phosphorus in CKD patients. Similarly, a cross-sectional study in older men demonstrated that higher levels of physical activity and a less sedentary lifestyle were associated with favorable kidney functions [46]. Increased physical activity by an extra hour was positively correlated with eGFR (β: 2.30, 95% CI: 1.46–3.14). However, increased sedentary duration by an extra hour was negatively associated with eGFR (β: −0.71, 95% CI: −1.08 to −0.35) in Type 2 diabetic patients [47].

Certain limitations and strengths should be taken into consideration when interpreting these results. First, this cross-sectional study design cannot clarify the causal relationship between NE and unhealthy lifestyle behaviors on kidney function parameters and cardiovascular risk factors. Second, the adherence or compliance of CKD patients for following the diet plan, which was recommended by the dietitian was not recorded in the “Integrated Chronic Disease Care Network” database. Thus, the present study could not estimate dietary total energy or protein intake in CKD patients from the database. Therefore, we could not clarify the influence of dietary intake on our findings. Finally, there was no information on compliance of participants following the diet plan provided or the recommendations for protein, phosphorous, potassium, sodium, and water in the database system. To the best of our knowledge, no prior studies have investigated the association of NE programs with blood lipid profiles in CKD patients. The present study is also the first study to explore the effects of NE and its interaction with lifestyle behaviors among CKD patients in clinical settings. Therefore, our findings provide new information that NE is not only beneficial to delay the progression of CKD but also ameliorate the abnormalities of the blood lipid profile in CKD patients. Additionally, our study used a large sample size from the clinical settings, which provides a more accurate interpretation of the results.

## 5. Conclusions

In conclusion, our study suggests that NE might serve as an effective supporting program to slow kidney function decline and cardiovascular risk factors in CKD patients. Additionally, the joint exposure of NE and healthy lifestyles potentially provides better self-health management for CKD patients. Future prospective studies focusing on education and lifestyle interventions in patients with early CKD stages or pre-ESRD are necessary to confirm our findings.

## Figures and Tables

**Table 1:** Characteristics of 2176 chronic kidney disease patients at stages 3–5 with or without nutrition education ^a^.

Characteristics	All (*n* = 2176)	Without Nutrition Education (*n* = 943)	With Nutrition Education (*n* = 1233)	*p* ^b^
Age (years)	72.0 (19.0)	75.0 (18.0)	70.0 (19.0)	<0.001
Gender				0.72
Male	1284 (59.0)	552 (58.5)	732 (59.4)	
Female	892 (41.0)	391 (41.5)	501 (40.6)	
Education				<0.001
<high school	1453 (66.8)	693 (73.5)	760 (61.6)	
≥high school	723 (33.2)	250 (26.5)	473 (38.4)	
Marital status				0.01
Unmarried	652 (30.0)	310 (32.9)	342 (27.7)	
Married	1524 (70.0)	633 (67.1)	891 (72.3)	
Smoking				0.24
No	1620 (74.4)	714 (75.7)	906 (73.5)	
Yes	556 (25.5)	229 (24.3)	327 (26.5)	
Drinking				0.51
No	1885 (86.6)	822 (87.2)	1063 (86.2)	
Yes	291 (13.4)	121 (12.8)	170 (13.8)	
Chewing betel nut				0.007
No	2097 (96.4)	922 (97.8)	1175 (95.3)	
Yes	79 (3.6)	21 (2.2)	58 (4.7)	
Physical activity ^c^				0.002
Inactive	1527 (70.2)	694 (73.6)	833 (67.6)	
Active	649 (29.8)	249 (26.4)	400 (32.4)	
Diuretic drugs user ^d^	541 (32.0)	248 (36.0)	293 (29.3)	0.004
Other drugs user ^e^	1871 (88.0)	764 (84.9)	1107 (90.3)	<0.001

^a^ Data are expressed as median (interquartile range) for continuous variables or number (percentage) for categorical variables.^b^ The *p* values were analyzed using the Wilcoxon rank-sum test for continuous variables and a 2-sided chi-square test for categorical variables.^c^ Physically active was defined as engaging in physical activity for ≥30 min/week.^d^ *n* = 1690 for all, *n* = 689 for without nutrition education, and *n* = 1001 for with nutrition education.^e^ Other drugs included angiotensin II receptor blocker, angiotensin-converting enzyme inhibitor, calcium channel blocker, calcium-phosphorus binder, iron supplementation, antihypertensive agent, hypolipidemic agent, hypoglycemic agent, and insulin injection. *n* = 2125 for all, *n* = 899 without nutrition education, and *n* = 1226 with nutrition education.

**Table 2:** Clinical and biochemical data of 2176 chronic kidney disease patients at stages 3–5 with or without nutrition education ^a^.

Characteristics	All (*n* = 2176)	Without Nutrition Education (*n* = 943)	With Nutrition Education (*n* = 1233)	*p* ^b^
BMI (kg/m^2^)	25.1 (5.3)	25.0 (5.1)	25.2 (5.5)	0.012
Systolic BP (mmHg)	133.0 (24.0)	132.0 (26.0)	134.0 (23.0)	0.28
Diastolic BP (mmHg)	72.0 (16.0)	71.0 (16.0)	72.0 (16.0)	0.5
FBG (mmol/L)	6.0 (2.3)	6.0 (2.5)	5.9 (2.1)	0.1
HbA_1c_ (%)	6.3 (1.6)	6.5 (1.8)	6.2 (1.5)	<0.001
Albumin (g/L)	42.0 (7.0)	41.0 (7.5)	42.0 (7.0)	0.002
Blood lipids				
TG (mmol/L)	1.4 (1.2)	1.5 (1.2)	1.4 (1.1)	0.075
TC (mmol/L)	4.8 (1.6)	4.8 (1.5)	4.8 (1.6)	0.89
HDL-C (mmol/L)	1.1 (0.5)	1.1 (0.5)	1.2 (0.5)	0.07
LDL-C (mmol/L)	2.5 (1.1)	2.6 (1.1)	2.4 (1.1)	0.005
Minerals				
C-Ca (mmol/L)	2.2 (0.1)	2.2 (0.2)	2.2 (0.1)	0.09
Phosphorus (mmol/L)	1.3 (0.4)	1.3 (0.4)	1.3 (0.3)	0.51
Kidney function				
BUN (mmol/L)	11.8 (9.3)	12.1 (12.9)	11.4 (7.9)	0.001
Serum creatinine (μmol/L)	202.9 (166.2)	207.6 (253.3)	200.0 (133.8)	0.04
eGFR (mL/min/1.73 m^2^)	29.2 (23.2)	27.8 (27.9)	30.2 (19.8)	0.003
Urinary protein				
Urine protein (g/L)	0.7 (1.7)	0.5 (1.4)	0.8 (1.8)	<0.001
Urine creatinine (mmol/L)	7.0 (5.7)	7.6 (6.2)	6.7 (5.7)	< 0.001
Urine protein to creatinine ratio (mg/mmol) ^c^	77.9 (219.3)	82.6 (222.2)	71.0 (199.8)	0.019
Chronic kidney disease stage, n (%)				<0.001
Stage 3a	272 (12.5)	163 (17.3)	109 (8.8)	
Stage 3b	779 (35.8)	264 (28.0)	515 (41.8)	
Stage 4	665 (30.6)	249 (26.4)	416 (33.7)	
Stage 5	460 (21.1)	267 (28.3)	193 (15.7)	

BMI: body mass index, BP: blood pressure, FBG: fasting blood glucose, HbA_1c_: glycated hemoglobin A_1c_, TG: triglycerides, TC: total cholesterol, HDL-C: high-density lipoprotein cholesterol, LDL-C: low-density lipoprotein cholesterol, C-Ca: corrected calcium, BUN: blood urea nitrogen, eGFR: estimated glomerular filtration rate. ^a^ Data are expressed as median (interquartile range) for continuous variables or number (percentage) for categorical variables. ^b^ The *p* values were analyzed using Wilcoxon rank-sum test for continuous variables and 2-sided chi-square test for categorical variables. ^c^ *n* = 1723 for all, *n* = 842 without nutrition education, and *n* = 881 with nutrition education.

**Table 3:** Adjusted beta (β) coefficients and 95% confidence intervals of kidney function parameters by nutrition education and its interaction with lifestyle factors in 2176 chronic kidney disease patients at stages 3–5 ^a^.

	BUN (mmol/L)	Serum Creatinine (μmol/L)	eGFR (mL/min/1.73 m^2^)	Urine Protein (g/L)	Urine Creatinine (mmol/L)	Urine PCR (mg/mmol)
With education	−4.16 (−5.14 to −3.19) **	−122.78 (−145.76 to −99.81) **	2.92 (1.55 to 4.30) **	−0.25 (−0.67 to 0.16)	−1.51 (−2.00 to −1.02) **	−40.21 (−74.2 to −6.22) *
**Nutrition education by smoking**						
*Ref: without education, smoking*						
Non-smoking	−0.01 (−1.84 to 1.84)	−55.66 (−98.99 to −12.33) *	2.32 (−0.28 to 4.92)	−0.16 (−0.95 to 0.63)	−0.83 (−1.76 to 0.11)	−70.68 (−131.72, −9.64) *
*With education*						
Non-smoking	−4.04 (−5.81 to −2.28) **	−162.07 (−203.56 to −120.59) **	4.92 (2.42 to 7.41) **	−0.42 (−1.17 to 0.33)	−2.04 (−2.94 to −1.15) **	−97.34 (−157.36 to −37.32) **
Smoking	−4.52 (−6.40 to −2.63) **	−168.79 (−213.13 to −124.44) **	3.83 (1.17 to 6.49) **	−0.24 (−1.04 to 0.56)	−2.33 (−3.27 to −1.38) **	−78.21 (−143.46 to −12.95) *
**Nutrition education by drinking**						
*Ref: without education, drinking*						
Non-drinking	1.79 (−0.50 to 4.07)	42.26 (−11.26 to 95.78)	−4.40 (−7.60 to −1.20) **	−0.42 (−1.38 to 0.55)	−2.02 (−3.17 to −0.88) **	1.92 (−72.35 to 76.18)
*With education*						
Non-drinking	−2.64 (−4.87 to −0.41) *	−83.92 (−136.15 to −31.69) **	4.31 (0.38 to 8.24) *	−0.69 (−1.63 to 0.25)	−3.24 (−4.36 to −2.13) **	−42.03 (−115.61 to, 31.55)
Drinking	−2.50 (−5.10 to 0.11)	−101.65 (−162.66 to −40.64) **	−0.79 (−4.44 to 2.86)	−0.13 (−1.22 to 0.97)	−3.27 (−4.57 to −1.98) **	−17.18 (−106.36 to 72.01)
**Nutrition education by physical activity**						
*Ref: without education, inactive*						
Active	−2.14 (−3.84 to −0.45) *	−64.77 (−104.52 to −25.02) **	3.63 (1.24 to 6.01) **	0.03 (−0.69 to 0.76)	1.39 (0.54 to 2.24) **	−45.14 (−100,04 to 9.76)
*With education*						
Inactive	−4.59 (−5.74 to −3.44) **	−140.25 (−167.33 to −113.18) **	3.67 (2.04 to 5.29) **	−0.20 (−0.69 to 0.29)	−0.95 (−1.52 to −0.37) **	−45.09 (−85.56 to −4.62) *
Active	−5.25 (−6.67 to −3.82) **	−144.47 (−177.98 to −110.96) **	4.73 (2.71 to 6.74) **	−0.36 (−0.97 to 0.24)	−1.47 (−2.18 to −0.76) **	−74.09 (−124.31 to −23.88) **

BUN: blood urea nitrogen, eGFR: estimated glomerular filtration rate, PCR: protein-to-creatinine ratio. ^a^ The model was adjusted for age, gender, education, marital status, occupation, smoking, drinking alcohol, chewing betel nut, physical activity, use of diuretics and other drugs, body mass index, systolic blood pressure, and diastolic blood pressure. * *p* < 0.05, ** *p* < 0.01.

**Table 4:** Adjusted odds ratios and 95% confidence intervals of cardiovascular risk factors by nutrition education and its interaction with lifestyle factors in 2176 chronic kidney disease patients at stages 3–5 ^a^.

	High FBG (≥5.6 mmol/L)	High HbA_1c_ (≥5.7%)	High TG (≥1.7 mmol/L)	High TC (≥5.2 mmol/L)	Low HDL-C (<1.04 mmol/L)	High LDL-C (≥2.6 mmol/L)	High C-Ca (≥2.4 mmol/L)	High P (≥1.5 mmol/L)
*n*	1400	1229	804	538	308	805	246	506
With education	0.88 (0.70–1.10)	0.61 (0.45–0.82) **	0.72 (0.57–0.89) **	0.97 (0.75–1.25)	0.68 (0.48–0.98) *	0.76 (0.60–0.96) *	0.81 (0.60–1.11)	0.76 (0.59–0.98) *
**Nutrition education by smoking**								
*Ref: without education, smoking*								
Non-smoking	1.23 (0.80–1.89)	0.93 (0.51–1.69)	0.97 (0.64–1.47)	0.91 (0.57–1.48)	0.73 (0.37–1.42)	1.00 (0.64–1.55)	0.58 (0.32–1.02)	0.70 (0.44–1.11)
*With education*								
Non-smoking	1.07 (0.72–1.61)	0.59 (0.33–1.04)	0.70 (0.47–1.03)	0.92 (0.59–1.46)	0.49 (0.26–0.92) *	0.81 (0.53–1.24)	0.55 (0.32–0.95) *	0.54 (0.35–0.84) **
Smoking	0.90 (0.58–1.39)	0.53 (0.30–0.96) *	0.72 (0.47–1.09)	0.89 (0.55–1.43)	0.71 (0.37–1.35)	0.62 (0.40–0.97) *	0.50 (0.27–0.94) *	0.72 (0.45–1.14)
RERI	−0.06 (−0.59–0.48)	0.13 (−0.41–0.67)	0.01 (−0.43–0.44)	0.12 (−0.38–0.62)	0.05 (−0.57–0.68)	0.19 (−0.22–0.61)	0.47 (0.09–0.85) *	0.12 (−0.30–0.54)
AP	−0.05 (−0.54–0.43)	0.22 (−0.78–1.22)	0.01 (−0.62–0.64)	0.13 (−0.44–0.70)	0.11 (−1.22–1.43)	0.24 (−0.32–0.80)	0.86 (−0.11–1.83)	0.23 (−0.61–1.06)
**Nutrition education by drinking**								
*Ref: without education, drinking*								
Non-drinking	0.87 (0.51–1.49)	0.80 (0.36–1.79)	1.21 (0.73–2.03)	0.95 (0.52–1.70)	1.27 (0.55–2.94)	0.67 (0.39–1.14)	1.67 (0.71–3.91)	1.31 (0.73–2.37)
*With education*								
Non-drinking	0.75 (0.44–1.26)	0.52 (0.24–1.13)	0.83 (0.50–1.37)	0.94 (0.53–1.67)	0.84 (0.37–1.89)	0.58 (0.32–1.05)	1.36 (0.59–3.14)	0.98 (0.55–1.75)
Drinking	1.00 (0.54–1.87)	0.40 (0.17–0.94) *	0.96 (0.53–1.73)	0.84 (0.43–1.65)	0.81 (0.55–2.94)	0.53 (0.32–0.90) *	0.85 (0.31–2.35)	0.85 (0.44–1.67)
RERI	−0.13 (−0.82–0.57)	0.31 (−0.27–0.89)	−0.34 (−1.10–0.41)	0.16 (−0.46–0.78)	−0.24 (−1.35–0.87)	0.28 (−0.14–0.71)	−0.16 (−1.34–1.01)	−0.19 (−0.97–0.60)
AP	−0.17 (−1.04–0.70)	0.60 (−0.91–2.11)	−0.41 (−1.19–0.36)	0.17 (−0.55–0.88)	−0.28 (−1.44–0.87)	0.53 (−0.47–1.54)	−0.12 (−0.92–0.68)	−0.19 (−0.92–0.54)
**Nutrition education by physical activity**								
*Ref: without education, inactive*								
Active	1.01 (0.68–1.50)	0.94 (0.55–1.61)	0.77 (0.52–1.12)	1.58 (1.00–2.49) *	0.95 (0.51–1.78)	0.74 (0.50–1.10)	0.66 (0.37–1.18)	0.59 (0.37–0.93) *
*With education*								
Inactive	0.87 (0.66–1.14)	0.58 (0.40–0.83) **	0.69 (0.53–0.90) **	1.05 (0.78–1.42)	0.74 (0.49–1.12)	0.72 (0.55–0.94) *	0.69 (0.48–0.99) *	0.70 (0.52–0.93) *
Active	0.90 (0.65–1.26)	0.64 (0.41–0.99) *	0.60 (0.43–0.83) **	1.27 (0.87–1.83)	0.54 (0.32–0.90) *	0.64 (0.46–0.90) **	0.86 (0.55–1.34)	0.58 (0.40–0.84) **
RERI	0.03 (−0.45–0.50)	0.12 (−0.42–0.66)	0.14 (−0.22–0.49)	−0.36 (−1.16–0.43)	−0.15 (−0.85–0.54)	0.19 (−0.18–0.55)	0.46 (0.01–0.90) *	0.30 (−0.06–0.65)
AP	0.03 (−0.50–0.56)	0.19 (−0.67–1.05)	0.23 (−0.37–0.82)	−0.29 (−0.93–0.36)	−0.29 (−1.57–0.99)	0.29 (−0.28–0.86)	0.51 (0.03–0.99) *	0.51 (−0.11–1.13)

FBG: fasting blood glucose, HbA_1c_: glycated hemoglobin A_1c_, TG: triglycerides, TC: total cholesterol, HDL-C: high-density lipoprotein cholesterol, LDL-C: low-density lipoprotein cholesterol, C-Ca: corrected calcium, P: phosphorus. ^a^ The model was adjusted for age, gender, education, marital status, occupation, smoking, drinking alcohol, chewing betel nut, physical activity, use of diuretics and other drugs, body mass index, systolic blood pressure, and diastolic blood pressure. * *p* < 0.05, ** *p* < 0.01.

## Data Availability

The data that support the findings of this study are available from “Integrated Chronic Kidney Disease Care Network,” but restricted for research use only. The data are not publicly available. Data are available from the authors upon reasonable request and with permission from the Department of Nephrology at Shuang Ho Hospital, Taipei Medical University, Taiwan.

## Supplementary Materials

The following are available online at https://www.mdpi.com/2072-6643/13/2/298/s1. Table S1: Adjusted beta (β) coefficients and 95% confidence intervals (CIs) of kidney function parameters by lifestyle factors. Table S2: Adjusted odds ratios (ORs) and 95% confidence intervals (CIs) of cardiovascular risk factors by lifestyle factors in 2176 chronic kidney disease patients at stages 3–5.
