# Supplementary material for: Association of Nutrition Education and Its Interaction with Lifestyle Factors on Kidney Function Parameters and Cardiovascular Risk Factors among Chronic Kidney Disease Patients in Taiwan
Source: Nutrients. 2021 Jan 21;13(2):298. doi: 10.3390/nu13020298 (PMC7909784; doi:10.3390/nu13020298)
Supplement: Supplementary file 1 [file nutrients-13-00298-s001.pdf]

Association of Nutrition Education and Its Interaction with Lifestyle Factors on Kidney Function Parameters and Cardiovascular Risk Factors among Chronic Kidney Disease Patients in Taiwan

Adi Lukas Kurniawan [1,2], Ya-Lan Yang [3], Mei-Yun Chin [3,*], Chien-Yeh Hsu [4,5], Rathi Paramastri [1], Hsiu-An Lee [6], Po-Yuan Ni [1] and Jane C-J Chao [1,5,7,*]

Table S1. Adjusted beta (β) coefficients and 95% confidence intervals (CIs) of kidney function parameters by lifestyle factors [a].

	BUN (mmol/L)	Serum Creatinine (μmol/L)	eGFR (mL/min/1.73 m²)	Urine Protein (g/L)	Urine Creatinine (mmol/L)	Urine PCR (mg/mmol)
Smoking status (Ref: smoking)						
Model 1	-0.42<br>(-1.45 to 0.61)	**-25.03**<br>**(-49.16 to -0.91)***	**1.97**<br>**(0.48 to 3.45)****	**-0.49**<br>**(-0.90 to -0.08)***	-0.14<br>(-0.68 to 0.41)	**-73.97**<br>**(-109.04 to -38.91)****
Model 2	0.21<br>(-1.08 to 1.51)	-19.79<br>(-50.73 to 11.15)	1.62<br>(-0.19 to 3.42)	-0.18<br>(-0.72 to 0.36)	-0.15<br>(-0.79 to 0.50)	-43.00<br>(-88.00 to 1.99)
Drinking status (Ref: drinking)						
Model 1	0.41<br>(-0.85 to 1.68)	9.17<br>(-20.40 to 38.75)	-0.49<br>(-2.32 to 1.33)	-0.43<br>(-0.93 to 0.07)	**-0.72**<br>**(-1.39 to -0.05)***	**-44.57**<br>**(-87.28 to -1.87)***
Model 2	0.53<br>(-1.04 to 2.10)	24.09<br>(-13.22 to 61.41)	-1.72<br>(-3.90 to 0.45)	-0.52<br>(-1.17 to 0.14)	**-0.83**<br>**(-1.61 to -0.05)***	-13.54<br>(-67.60 to 40.52)
Physical activity (Ref: inactive)						
Model 1	**-2.10**<br>**(-3.00 to -1.20)****	**-44.46**<br>**(-65.57 to -23.36)****	**3.41**<br>**(2.11 to 4.71)****	-0.26<br>(-0.61 to 0.10)	0.31<br>(-0.16 to 0.79)	**-60.07**<br>**(-90.25 to -29.88)****
Model 2	**-1.34**<br>**(-2.41 to -0.27)***	**-30.48**<br>**(-56.00 to -4.96)***	**2.11**<br>**(0.62 to 3.60)****	-0.10<br>(-0.55 to 0.34)	0.13<br>(-0.40 to 0.66)	**-37.73**<br>**(-74.65 to -0.82)***

BUN: blood urea nitrogen, eGFR: estimated glomerular filtration rate, PCR: protein to creatinine ratio.

[a] Model 1 was adjusted for age and gender. Model 2 was adjusted for age, gender, education, marital status, occupation, smoking, drinking alcohol, chewing betel nut, physical activity, use of diuretics and other drugs, body mass index, systolic blood pressure, and diastolic blood pressure.

* $p < 0.05$, ** $p < 0.01$.

Table S2. Adjusted odds ratios (ORs) and 95% confidence intervals (CIs) of cardiovascular risk factors by lifestyle factors [a].

	**High FBG (≥ 5.6 mmol/L)**	**High HbA1c (≥ 5.7%)**	**High TG (≥ 1.7 mmol/L)**	**High TC (≥ 5.2 mmol/L)**	**Low HDL-C (< 1.04 mmol/L)**	**High LDL-C (≥ 2.6 mmol/L)**	**High C-Ca (≥ 2.4 mmol/L)**	**High P (≥ 1.5 mmol/L)**
Smoking status (Ref: smoking)								
Model 1	0.99 (0.79-1.25)	1.18 (0.88-1.58)	**0.78 (0.62-0.98)***	0.97 (0.74-1.27)	0.72 (0.51-1.02)	1.08 (0.84-1.37)	0.86 (0.60-1.23)	**0.70 (0.54-0.92)****
Model 2	1.21 (0.90-1.62)	1.03 (0.70-1.51)	0.97 (0.73-1.29)	0.99 (0.71-1.38)	0.70 (-0.45-1.09)	1.18 (0.87-1.59)	0.82 (0.54-1.26)	0.73 (0.52-1.01)
Drinking status (Ref: drinking)								
Model 1	0.77 (0.57-1.02)	1.27 (0.90-1.79)	0.82 (0.63-1.09)	0.85 (0.62-1.18)	0.94 (0.62-1.43)	0.82 (0.61-1.09)	1.45 (0.88-2.37)	0.93 (0.67-1.28)
Model 2	0.79 (0.55-1.31)	1.13 (0.73-1.76)	0.98 (0.69-1.39)	1.05 (0.70-1.57)	1.09 (0.64-1.87)	0.81 (0.57-1.15)	1.62 (0.91-2.91)	1.21 (0.81-1.80)
Physical activity (Ref: inactive)								
Model 1	0.97 (0.79-1.19)	0.81 (0.63-1.04)	**0.80 (0.65-0.97)***	**1.32 (1.01-1.74)***	**0.71 (0.52-0.98)***	0.90 (0.73-1.10)	0.94 (0.70-1.27)	**0.64 (0.51-0.82)****
Model 2	1.02 (0.80-1.31)	1.05 (0.77-1.42)	0.82 (0.65-1.04)	1.16 (0.92-1.47)	0.79 (0.54-1.14)	0.83 (0.65-1.06)	0.97 (0.69-1.37)	**0.73 (0.55-0.96)***

FBG: fasting blood glucose, HbA1c: glycated hemoglobin A1c, TG: triglycerides, TC: total cholesterol, HDL-C: high-density lipoprotein cholesterol, LDL-C: low-density lipoprotein cholesterol, C-Ca: corrected calcium, P: phosphorus.

[a] Model 1 was adjusted for age and gender. Model 2 was adjusted for age, gender, education, marital status, occupation, smoking, drinking alcohol, chewing betel nut, physical activity, use of diuretics and other drugs, body mass index, systolic blood pressure, and diastolic blood pressure.

* $p < 0.05$, ** $p < 0.01$.
